# Patterns of Obesity and Overweight in the Iranian Population: Findings of STEPs 2016

**DOI:** 10.3389/fendo.2020.00042

**Published:** 2020-02-26

**Authors:** Shirin Djalalinia, Sahar Saeedi Moghaddam, Ali Sheidaei, Nazila Rezaei, Seyed Sina Naghibi Iravani, Mitra Modirian, Hossein Zokaei, Moein Yoosefi, Kimiya Gohari, Ahmad Kousha, Zhaleh Abdi, Shohreh Naderimagham, Ahmad Reza Soroush, Bagher Larijani, Farshad Farzadfar

**Affiliations:** ^1^Deputy of Research and Technology, Ministry of Health and Medical Education, Tehran, Iran; ^2^Non-Communicable Diseases Research Center, Endocrinology and Metabolism Population Sciences Institute, Tehran University of Medical Sciences, Tehran, Iran; ^3^Department of Biostatistics, Faculty of Paramedical Sciences, Shahid Beheshti University of Medical Sciences, Tehran, Iran; ^4^Department of Biostatistics, Faculty of Medical Sciences, Tarbiat Modares University, Tehran, Iran; ^5^Department of Health Education and Promotion, Faculty of Health Sciences, Tabriz University of Medical Sciences, Tabriz, Iran; ^6^Department of Research and Education, National Institute of Health Research, Tehran University of Medical Sciences, Tehran, Iran; ^7^Obesity and Eating Habits Research Center, Endocrinology and Metabolism Molecular-Cellular Sciences Institute, Tehran University of Medical Sciences, Tehran, Iran; ^8^Endocrinology and Metabolism Research Center, Endocrinology and Metabolism Clinical Sciences Institute, Tehran University of Medical Sciences, Tehran, Iran

**Keywords:** obesity, overweight, BMI, STEPs, Iran

## Abstract

**Background:** Obesity has become a common health problem all over the world. Benefiting from a national representative sample, the present study aimed to estimate the prevalence of overweight/obesity and the distribution of Body Mass Index (BMI) levels in the Iranian adult population, by sex, age, and geographical distribution.

**Methods:** This was a large-scale national cross-sectional study of Non-communicable Diseases risk factor surveillance in Iran. Through a systematic random sampling cluster, 31,050 Iranian adult participants aged 18 years and over were enrolled in the study. The main research tools were used to assess three different levels of data, namely: (1) demographic, epidemiologic, and risk-related behavioral data, (2) physical measurements, and (3) lab measurements. Anthropometric measurements were taken using standard protocols and calibrated instruments.

**Results:** In 2016, the national prevalence rates of normal weight, obesity, and overweight/obesity among Iranian adults were, 36.7% (95% CI: 36.1–37.3), 22.7% (22.2–23.2), and 59.3% (58.7–59.9), respectively. There was a significant difference between the prevalence of obesity among males [15.3% (14.7–15.9)] and females [29.8% (29.0–30.5)] (*p* < 0.001). The 55–64 [31.5% (30.1–33.0)] and the 18–24 [8.3% (7.3–9.4)] year-old age groups had the highest and lowest prevalence of obesity, respectively. The results show a geographical pattern at provincial level, where the level of BMI increases among populations ranging from the southeastern to the northwestern regions of the country. The highest provincial prevalence of obesity was almost 2.5-fold higher than the lowest provincial prevalence.

**Conclusion:** We found a significant difference between the prevalence of obesity in males and females. Moreover, there was a considerable difference in the geographical pattern of the prevalence of obesity and overweight. Further evidence is warranted to promote strategies and interventions related to prevention and control of factors that are associated with weight gain.

## EBM Ratings Will Be Based on a Scale of 1 to 5, as Follows

Level I: Evidence obtained from at least one properly designed randomized controlled trial, systematic review and meta-analysis, and experimental study.
Level II: Evidence obtained from well-designed controlled trials without randomization.
Level III: Evidence obtained from well-designed cohort or case-control analytic studies.
Level IV: Evidence obtained from multiple time series with or without intervention, such as case studies. Dramatic results in uncontrolled trials might also be regarded as this type of evidence.
Level V: Opinions of respected authorities, based on descriptive studies, narrative reviews, clinical experience, or reports of expert committees.

The present paper reveals the most updated results of overweight/obesity and BMI at national and subnational levels in 2016. Using proportional to size sampling, one of the main strengths of the study was its comprehensive protocols focused on standards and regulatory guidelines.

## Introduction

Obesity has become a common health problem, and its prevalence as a global pandemic continues to increase in many developed and developing countries (1–3). According to recent analyses, obesity is probably the most important of the four prominent global risk factors that fulfill the criteria for being governmental health priorities.

It has been estimated that worldwide, 603.7 million adults (overall prevalence of 12.0%) were obese in 2015 (4). Compared to 1980, this prevalence has doubled in 73 countries (4). In 2015, globally, a burden of about 4.0 million deaths (7.1% of all deaths) and 120 million disability-adjusted life years (DALYs) (4.9% of all DALYs) were attributed to overweight and obesity (4).

In 2005, the estimated mean number of deaths attributable to excess Body Mass Index (BMI) in Iranian males and females were 13,000 and 17,000, respectively (5).

Based on the results of a systematic review which included evidence from January 2005 to January 2014, the range of overweight and obesity prevalence among the Iranian adult population at subnational level was between 12.8–76.4 and 2.4–35.4%, respectively (6).

The STEPs survey in 2011 showed that, overall, 22.3% of Iranian adults aged ≥15 years were obese (14% of males and 27.7% of females) (7). In 2011, Population Attributable Fraction (PAF) analysis showed that at least 33.78, 10.25, and 30.56% of the prevalent diabetes mellitus (DM) could be attributed to overweight (BMI ≥ 25 kg/m^2^), general obesity (BMI ≥ 30 kg/m^2^), and central obesity (waist circumference ≥ 90 cm), respectively (8). However, we have no new information following the STEPs survey of 2011 (9).

The World Health Organization (WHO) Action Plan for the control and prevention of Non-communicable Diseases (NCDs) and Sustainable Development Goals (SDGs target 3.4) requests member states to reduce the unconditional probability of deaths due to NCDs for people aged 30–70 years by modifying lifestyle and metabolic risk factors, including obesity and overweight. Based on the global agenda of risk reduction, having reliable information on the level, trend, and distribution of NCD risk factors—including overweight and obesity—is crucial for designing, implementing, and evaluating National Action Plans at country level (1, 10, 11).

Benefiting from national and subnational representative samples of STEPs survey 2016 (9), the present study estimates the prevalence of overweight/obesity and the distribution of BMI levels among the Iranian population, by sex, age, and geographical distribution.

## Methods

Following the WHO STEPwise approach to NCD risk factor surveillance, we designed and conducted the national STEPs survey 2016, with representative samples from urban and rural areas of Iran (12). The details of the procedures and methods of STEPs 2016 are discussed elsewhere (9). Here, we only point out some essential requirements.

### Sampling

We used the national postal code database, which includes addresses of all residential buildings in the country, as the sampling frame. Through systematic proportional to size cluster sampling, we selected proportional to size samples from rural and urban areas within each province. In sum, 3,105 clusters and 31,050 participants were selected from 18 ≤ year-old Iranian adults. The main research tools were used to assess three different levels of data, namely: (1) demographic, epidemiologic, and risk–related behavioral data, (2) physical measurements, (3) lab measurements (12–14). After collecting information from each household regarding age, sex, and other demographic characteristics of every member, we included the individuals of each household whose age was 18 years and above. We randomly excluded one or more individuals if a household had two or more individuals in the same age-sex group (age groups were 18–24, 25–29, 30–34, 35–39, 40–44, 45–49, 50–54, 55–59, 60–64, 65–69, 70, and above). We also excluded those with severe physical and mental conditions, which made them unable to be interviewed. For all included participants, we filled a questionnaire, measured anthropometric indices, and took blood and urine samples for lab tests (lab tests were done for those aged 25 years and above). The full details of the sampling protocol as well as sample size calculation have been fully described elsewhere (9).

### Questionnaire

Following the WHO STEPwise approach to risk factor surveillance, trained experts were involved in the processes of sampling and examinations. Information was recorded through validated questionnaires containing: demographic characteristics, lifestyle information (e.g., nutrition, physical activity, smoking, and alcohol consumption), history of metabolic risk factors and treatment, history of injuries and their risk factors, health care utilization, and screening programs (9).

### Physical Measurements

A team of trained healthcare experts performed the examinations under standard protocols using calibrated instruments (9). Systolic and diastolic blood pressures were measured thrice at a time interval of 5 min, and the mean values of the second and third instances were used for further analyses. Weight was measured in light clothing to the nearest 100 g, and height was measured to the nearest 1 cm while the participants were standing, without shoes, with shoulders in a normal position. The BMI of each individual was calculated as weight (kg) divided by squared height (m^2^). The WHO criteria were used, so that individuals with BMI < 18.5, 18.5 ≤ BMI < 25, 25 ≤ BMI < 30, 30 ≤ BMI were considered underweight, normal, overweight, and obese, respectively. Obese individuals were divided into class I obese (30 ≤ BMI < 35), class II obese (35 ≤ BMI < 40), and class III obese (40 ≤ BMI) (1, 15).

### Laboratory Measurements

The collected blood and urine samples were stored under a temperature of 4°C in vaccine transfer boxes. They were transferred in <18 h as the shortest possible time—to the central processing and archiving laboratory of study in the Non-Communicable Diseases Research Center (NCDRC) of the Endocrinology and Metabolism Population Sciences Institute of Tehran University of Medical Sciences. All the collected samples were examined by unique brands of devices and kits in the NCDRC laboratory (9).

### Definitions of Variables

Education was defined as the number of successfully completed years of schooling and was categorized into four subgroups [0 (Illiterate), 1–6, 7–12, and >12 years]. Principal component analysis was used to calculate the participants' wealth index from household asset data. The participants' calculated wealth indices were categorized into five quintiles from the poorest (first quintile) to the richest (fifth quintile). Diabetes based on Fasting Plasma Glucose (FPG) was defined as FPG ≥ 126 mg/dl or self-reported [based on the intake of Oral Hypoglycemic Agents) OHA and/or insulin injection]. Diabetes based on HbA1c was defined as HbA1c ≥ 6.4% or self-reported (OHA and/or insulin injection). Pre-diabetes based on FPG was defined as 100 ≤ FPG < 126 mg/dl among those who were not recognized as diabetic. Pre-diabetes based on HbA1c was defined as 5.7 ≤ HbA1c < 6.4% among those who were not recognized as diabetic. Hypertriglyceridemia was defined as serum triglycerides ≥ 150 mg/dl. LDL–C was considered as a level of low-density lipoprotein cholesterol ≥ 100 mg/dl. Hypercholesterolemia was defined as total cholesterol ≥ 200 mg/dl or self-reported drug intake. Hypertension was defined as a systolic blood pressure ≥ 140 mmHg, or a diastolic blood pressure ≥ 90 mmHg, or self-reported drug intake. Pre-hypertension was defined as 120 ≤ systolic blood pressure < 140 mmHg or 80 ≤ diastolic blood pressure < 90 mmHg among those who were not recognized as hypertensive. Participants were considered as “ever tobacco smokers” if they reported an experience of any tobacco derivatives during their lifetime. “Ever daily cigarette smokers” and “current daily cigarette smokers” were defined, respectively, as those with an experience of cigarette smoking on a daily basis during their lifetime, and currently smoking on a daily basis. The incidence of heart attack and stroke were defined as self-reported histories of events within the past year.

### Statistical Analyses

The data were used for presenting the descriptive statistics of interested variables, by sex and age subgroups at national and subnational levels. Moreover, the specifications of geographical data sampling enabled us to provide the interest outcomes at provincial level.

The national prevalence rates and means (for all ages) have been presented with 95% Confidence Intervals (95% CI) in the tables. Age-standardization of provincial mean BMI and prevalence of each BMI for defined categories was achieved according to the 2016 National Population and Housing Census conducted by Iran's Statistical Center^1^. The aforementioned results have been presented in maps, with a combination of area of residence and sex.

While assessing laboratory indices with different BMI categories, individuals who were 25 years and older were taken into consideration. The age-adjusted Odds Ratio (OR) of BMI categories with respect to smoking status, anthropometry (pre-hypertension and hypertension), and laboratory (pre-diabetes, diabetes, LDL-C, hypertriglyceridemia, hypercholesterolemia) and self-reported incidence of cardiovascular diseases (heart attack and stroke) variables were calculated by logistic regression at three significance levels. These analyses were performed by Stata software (version 11) and R software (version 3.0.2).

### Ethical Considerations

Ethical approval for the study was obtained from the Ethical Committee of the National Institute for Medical Research Development (NIMAD) (ID: IR.NIMAD.REC.1394.032). Participation in the study was voluntary. Regarding ethical considerations, the objectives and methods of the study were described for all eligible individuals and written informed consent was obtained from all those who accepted to participate.

## Results

In the STEPs study, of the 30,541 participants who completed the questionnaires (step 1), 30,042 individuals were anthropometrically measured (step 2). In the current study, we included 29,124 participants (15,044 females and 14,080 males) who had non-missing BMI values. Most of the participants were urban residents (71.2%), aged 25–34 years (23.7%), educated (14.6% were illiterate), married (76.6%), and were covered by basic insurance (91.9%). The demographic characteristics of the participants are presented in Table 1.

**Table 1:** Demographic characteristics of participants.

**Variable**	**Characteristics**	**Sex**		
		**Female *n* = 15,044**	**Male *n* = 14,080**	**Both *n* = 29,124**
Area of residency	Rural	4,467 (14.9%)	4,118 (14.0%)	8,585 (28.8%)
	Urban	10,577 (36.6%)	9,962 (34.6%)	20,539 (71.2%)
Age category	18–24 years	1,420 (4.9%)	1,231 (4.5%)	2,651 (9.4%)
	25–34 years	3,529 (12.1%)	3,328 (11.6%)	6,857 (23.7%)
	35–44 years	3,203 (11.0%)	3,048 (10.5%)	6,251 (21.5%)
	45–54 years	2,867 (9.7%)	2,571 (8.7%)	5,438 (18.4%)
	55–64 years	2,198 (7.5%)	2,025 (6.9%)	4,223 (14.4%)
	65–69 years	759 (2.6%)	616 (2.1%)	1,375 (4.7%)
	70 years and more	1,068 (3.6%)	1,261 (4.3%)	2,329 (7.9%)
Education	Illiterate	2,959 (9.9%)	1,400 (4.7%)	4,359 (14.6%)
	1–6 years	3,938 (13.3%)	3,390 (11.5%)	7,328 (24.8%)
	7–12 years	5,392 (18.8%)	6,202 (21.7%)	11,594 (40.5%)
	More than 12 years	2,755 (9.5%)	3,088 (10.7%)	5,843 (20.2%)
Marital status	Never married	1,937 (6.8%)	2,411 (8.8%)	4,348 (15.6%)
	Married	11,008 (37.7%)	11,338 (38.9%)	22,346 (76.6%)
	Divorced/separated	401 (1.4%)	116 (0.4%)	517 (1.8%)
	Widowed	1,619 (5.6%)	147 (0.5%)	1,766 (6.1%)
Basic insurance	No	998 (3.5%)	1,271 (4.6%)	2,269 (8.1%)
	Yes	14,011 (48.0%)	12,784 (44.0%)	26,795 (91.9%)
Type of basic insurance	Health insurance	5,599 (19.1%)	5,265 (18.2%)	10,864 (37.3%)
	Social insurance	6,307 (21.8%)	5,769 (19.9%)	12,076 (41.7%)
	Army insurance	611 (2.1%)	563 (2.0%)	1,174 (4.1%)
	Imam Khomeini Relief Foundation insurance	131 (0.5%)	47 (0.2%)	178 (0.6%)
	Other insurances	1,363 (4.5%)	1,140 (3.8%)	2,503 (8.2%)
	No insurance	998 (3.5%)	1,271 (4.6%)	2,269 (8.1%)
Complementary insurance	No	11,715 (40.4%)	11,117 (38.7%)	22,832 (79.0%)
	Yes	3,174 (11.0%)	2,860 (10.0%)	6,034 (21.0%)
Wealth index quintile	Poorest	3,019 (10.4%)	2,616 (9.2%)	5,635 (19.6%)
	2	2,988 (10.6%)	2,644 (9.4%)	5,632 (19.9%)
	3	2,919 (10.3%)	2,757 (9.7%)	5,676 (20.0%)
	4	2,868 (10.1%)	2,818 (10.0%)	5,686 (20.1%)
	Richest	2,825 (10.1%)	2,906 (10.4%)	5,731 (20.4%)

*Data presented as number and percent (%)*.

*All values were calculated among those with a non-missing variable*.

In 2016, the national prevalence of normal weight, obesity, and overweight/obesity in 18≤ year-old Iranian adults was estimated at, 36.7% (95% CI: 36.1–37.3), 22.7% (22.2–23.2), and 59.3% (58.7–59.9), respectively. In the obese group, the distribution of the three obesity categories were estimated at 16.9% (16.4–17.3) in class I obese, 4.6% (4.3–4.9) in class II obese, and 1.3% (1.1–1.4) in class III obese (Table 2). With respect to differences in sex, there was a significant difference between the prevalence of obesity in males [15.3% (14.7–15.9)] and females [29.8% (29.0–30.5)] (*p* < 0.001). This difference was also detected in the national prevalence rate of overweight/obesity [M: 53.6% (52.7–54.4), F: 64.7% (63.9–65.5) (*p* < 0.001)] (Table 2). Compared to females, Iranian males had a lower mean BMI [25.6 (25.5–25.7) vs. 27.4 (27.3–27.5) kg/m^2^] (*p* < 0.001). Another noticeable point is that 4.3% (4.0–4.6) of males and 3.7% (3.4–4.0) of females were underweight (Table 2).

**Table 2:** Distribution of different categories of BMI prevalence (%) according to selected characteristics of Iranian adults.

**Variable**	**Characteristics**	**Underweight** **(BMI < 18.5)**	**Normal** **(18.5 ≤ BMI <25)**	**Overweight** **(25 ≤ BMI <30)**	**Overweight/obesity** **(25 ≤ BMI)**	**Obesity** **(30 ≤ BMI)**	**Class I obesity** **(30 ≤ BMI < 35)**	**Class II obesity** **(35 ≤ BMI < 40)**	**Class III obesity** **(40 ≤ BMI)**
Overall		4.0 (3.8–4.2)	36.7 (36.1–37.3)	36.6 (36.0–37.1)	59.3 (58.7–59.9)	22.7 (22.2–23.2)	16.9 (16.4–17.3)	4.6 (4.3–4.9)	1.3 (1.1–1.4)
Area of residency	Rural	6.2 (5.7–6.8)	42.2 (41.1–43.2)	32.1 (31.1–33.1)	51.6 (50.5–52.7)	19.5 (18.6–20.4)	14.5 (13.7–15.3)	4.0 (3.6–4.5)	1.0 (0.8–1.2)
	Urban	3.1 (2.8–3.3)	34.5 (33.8–35.2)	38.4 (37.7–39.1)	62.4 (61.7–63.1)	24.1 (23.4–24.7)	17.9 (17.3–18.4)	4.8 (4.5–5.1)	1.4 (1.2–1.5)
Sex	Female	3.7 (3.4–4.0)	31.6 (30.8–32.4)	35.0 (34.2–35.8)	64.7 (63.9–65.5)	29.8 (29.0–30.5)	20.9 (20.2–21.6)	6.8 (6.4–7.3)	2.0 (1.8–2.2)
	Male	4.3 (4.0–4.6)	42.1 (41.3–43.0)	38.3 (37.4–39.1)	53.6 (52.7–54.4)	15.3 (14.7–15.9)	12.6 (12.0–13.2)	2.2 (2.0–2.5)	0.4 (0.3–0.6)
Age category	18–24 years	10.8 (9.6–12.0)	59.2 (57.3–61.2)	21.6 (20.0–23.3)	30.0 (28.2–31.8)	8.3 (7.3–9.4)	6.5 (5.6–7.5)	1.6 (1.1–2.1)	0.2 (0.1–0.4)
	25–34 years	5.4 (4.8–5.9)	46.7 (45.4–47.9)	34.1 (32.9–35.3)	48.0 (46.7–49.2)	13.9 (13.0–14.7)	10.9 (10.2–11.7)	2.3 (1.9–2.6)	0.7 (0.5–0.9)
	35–44 years	2.3 (1.9–2.6)	33.0 (31.8–34.3)	39.4 (38.1–40.6)	64.7 (63.5–66.0)	25.4 (24.2–26.5)	19.1 (18.1–20.1)	4.9 (4.4–5.5)	1.3 (1.0–1.6)
	45–54 years	1.9 (1.5–2.3)	25.9 (24.7–27.0)	41.1 (39.7–42.4)	72.2 (71.0–73.5)	31.2 (29.9–32.4)	22.1 (21.0–23.3)	7.0 (6.2–7.7)	2.1 (1.7–2.5)
	55–64 years	2.5 (2.0–3.0)	26.4 (25.0–27.7)	39.6 (38.1–41.1)	71.2 (69.7–72.6)	31.5 (30.1–33.0)	22.3 (21.0–23.6)	7.6 (6.7–8.4)	1.7 (1.3–2.1)
	65–69 years	2.4 (1.6–3.2)	27.9 (25.4–30.4)	40.2 (37.5–42.9)	69.7 (67.2–72.2)	29.5 (27.0–32.0)	22.6 (20.3–24.9)	4.9 (3.7–6.0)	2.0 (1.3–2.8)
	70 years and more	5.0 (4.1–5.9)	39.5 (37.4–41.5)	35.9 (33.9–37.9)	55.5 (53.4–57.6)	19.6 (17.9–21.2)	15.6 (14.1–17.2)	3.1 (2.4–3.9)	0.8 (0.4–1.2)
Education	Illiterate	4.6 (3.9–5.2)	33.5 (32.1–35.0)	34.3 (32.8–35.8)	61.9 (60.4–63.4)	27.6 (26.2–29.0)	19.4 (18.2–20.6)	6.6 (5.8–7.4)	1.6 (1.2–2.0)
	1–6 years	3.6 (3.2–4.1)	31.3 (30.2–32.4)	36.6 (35.4–37.7)	65.1 (63.9–66.2)	28.5 (27.4–29.6)	20.5 (19.5–21.4)	6.2 (5.6–6.8)	1.8 (1.5–2.1)
	7–12 years	3.9 (3.5–4.3)	38.0 (37.0–38.9)	37.1 (36.1–38.0)	58.2 (57.2–59.1)	21.1 (20.3–21.9)	16.0 (15.3–16.7)	4.0 (3.6–4.4)	1.1 (0.9–1.3)
	More than 12 years	4.2 (3.7–4.7)	43.1 (41.8–44.5)	37.2 (36.0–38.5)	52.7 (51.3–54.0)	15.4 (14.5–16.4)	12.5 (11.6–13.4)	2.3 (1.9–2.7)	0.6 (0.4–0.8)
Marital status	Never married	9.2 (8.3–10.1)	56.0 (54.4–57.5)	25.8 (24.4–27.1)	34.8 (33.3–36.3)	9.0 (8.1–9.9)	7.3 (6.5–8.1)	1.4 (1.1–1.8)	0.3 (0.1–0.4)
	Married	2.9 (2.7–3.1)	33.5 (32.9–34.1)	39.0 (38.3–39.7)	63.6 (62.9–64.2)	24.6 (24.0–25.2)	18.3 (17.8–18.9)	4.9 (4.6–5.2)	1.3 (1.2–1.5)
	Divorced/separated	4.9 (3.1–6.8)	36.7 (32.4–41.0)	33.4 (29.1–37.6)	58.4 (54.0–62.8)	25.0 (21.1–28.9)	18.3 (14.8–21.8)	5.8 (3.8–7.9)	0.9 (0.0–1.8)
	Widowed	3.8 (2.9–4.7)	27.6 (25.3–29.9)	34.9 (32.6–37.2)	68.6 (66.2–71.0)	33.7 (31.4–36.0)	22.6 (20.6–24.7)	8.3 (6.9–9.8)	2.7 (1.9–3.5)
Basic insurance	No	4.0 (3.2–4.9)	43.2 (41.0–45.3)	33.2 (31.2–35.2)	52.8 (50.6–54.9)	19.6 (17.9–21.3)	14.7 (13.2–16.2)	3.8 (3.0–4.6)	1.1 (0.6–1.5)
	Yes	4.0 (3.7–4.2)	36.1 (35.5–36.7)	36.9 (36.3–37.5)	59.9 (59.3–60.5)	23.0 (22.5–23.5)	17.1 (16.6–17.5)	4.7 (4.4–4.9)	1.3 (1.1–1.4)
Type of basic insurance	Health insurance	5.5 (5.1–6.0)	39.2 (38.3–40.2)	34.3 (33.4–35.3)	55.2 (54.3–56.2)	20.9 (20.1–21.7)	15.4 (14.7–16.2)	4.4 (4.0–4.8)	1.1 (0.9–1.3)
	Social insurance	2.7 (2.4–3.0)	34.0 (33.2–34.9)	38.8 (37.9–39.7)	63.3 (62.4–64.2)	24.5 (23.7–25.2)	18.2 (17.5–18.9)	4.9 (4.5–5.3)	1.3 (1.1–1.5)
	Army insurance	2.6 (1.7–3.5)	32.2 (29.5–35.0)	39.7 (36.8–42.5)	65.2 (62.4–68.0)	25.5 (23.0–28.1)	18.3 (16.0–20.5)	5.2 (3.9–6.5)	2.0 (1.2–2.9)
	Imam Khomeini Relief Foundation insurance	6.7 (2.9–10.5)	39.0 (31.6–46.4)	27.3 (20.5–34.1)	54.3 (46.7–61.9)	27.0 (20.0–33.9)	20.4 (14.0–26.7)	4.0 (1.0–7.0)	2.6 (0.1–5.1)
	Other insurances	3.9 (3.1–4.7)	34.3 (32.3–36.2)	38.2 (36.2–40.1)	61.8 (59.9–63.8)	23.7 (22.0–25.4)	17.8 (16.3–19.3)	4.5 (3.7–5.3)	1.4 (0.9–1.9)
	No insurance	4.0 (3.2–4.9)	43.2 (41.0–45.3)	33.2 (31.2–35.2)	52.8 (50.6–54.9)	19.6 (17.9–21.3)	14.7 (13.2–16.2)	3.8 (3.0–4.6)	1.1 (0.6–1.5)
Complementary insurance	No	4.5 (4.2–4.8)	38.6 (37.9–39.2)	35.5 (34.9–36.2)	56.9 (56.3–57.6)	21.4 (20.9–22.0)	16.0 (15.5–16.5)	4.4 (4.1–4.6)	1.1 (0.9–1.2)
	Yes	2.1 (1.7–2.5)	29.5 (28.3–30.7)	40.7 (39.5–42.0)	68.4 (67.2–69.6)	27.7 (26.5–28.8)	20.3 (19.3–21.4)	5.4 (4.8–5.9)	2.0 (1.6–2.4)
Wealth index quintile	Poorest	8.7 (8.0–9.5)	45.9 (44.6–47.3)	30.4 (29.2–31.6)	45.3 (44.0–46.7)	14.9 (14.0–15.9)	10.9 (10.1–11.8)	3.2 (2.7–3.6)	0.9 (0.6–1.1)
	2	3.8 (3.3–4.3)	37.7 (36.4–39.0)	35.0 (33.7–36.3)	58.5 (57.1–59.8)	23.5 (22.3–24.7)	17.2 (16.2–18.2)	4.8 (4.2–5.4)	1.5 (1.2–1.9)
	3	2.9 (2.5–3.3)	35.1 (33.8–36.4)	36.6 (35.3–37.9)	62.0 (60.7–63.4)	25.4 (24.2–26.6)	18.7 (17.6–19.7)	5.5 (4.9–6.1)	1.3 (1.0–1.6)
	4	2.8 (2.4–3.3)	33.4 (32.2–34.7)	38.3 (37.0–39.6)	63.7 (62.4–65.0)	25.4 (24.2–26.6)	19.1 (18.0–20.1)	5.1 (4.5–5.7)	1.2 (0.9–1.5)
	Richest	2.0 (1.7–2.4)	31.4 (30.1–32.6)	42.3 (40.9–43.6)	66.6 (65.3–67.8)	24.3 (23.2–25.5)	18.4 (17.4–19.4)	4.5 (3.9–5.0)	1.4 (1.1–1.8)

*Data in parentheses are 95% Confidence Intervals (CI)*.

Given the comparative results of the age groups, the highest and lowest prevalence of obesity belonged to the 55–64 [31.5% (30.1–33.0)] and 18–24 [8.3% (7.3–9.4)] year-olds, respectively. In the overweight/obese group, the highest and the lowest estimates belonged to the age groups of 45–54 [72.2% (71.0–73.5)] and 18–24 [30.0% (28.2–31.8)], respectively (Table 2).

The analysis of results showed that participants with >12 years of schooling had a significantly lower prevalence of obesity and overweight (*p* < 0.001). With regards to the marriage status, the never married population had the lowest prevalence of both obesity [9.0% (8.1–9.9)] and overweight [34.8% (33.3–36.3)] among all the age groups (Table 2).

**Figure 1:**
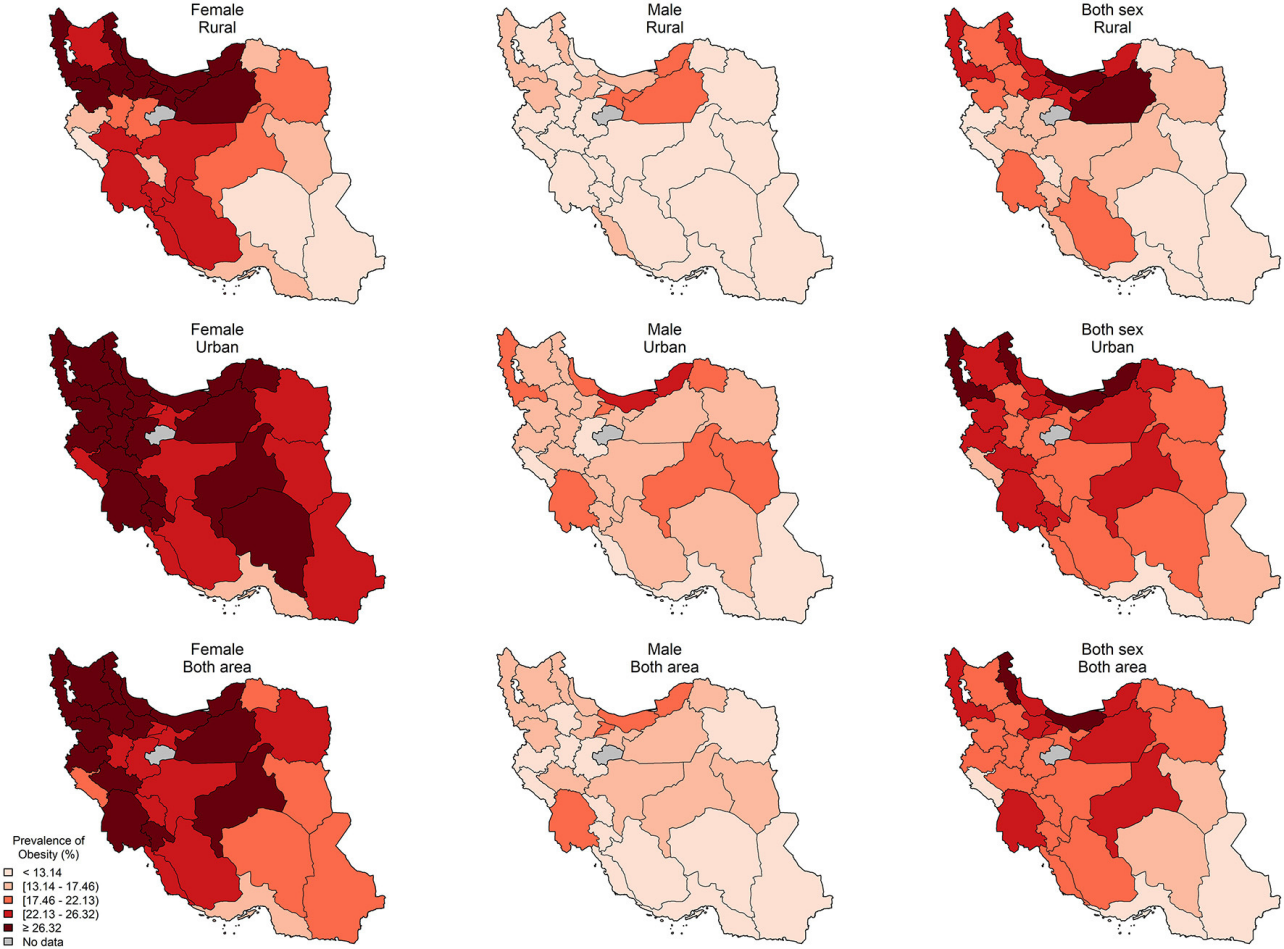
The provincial distribution of age-standardized prevalence of obesity (%) by residential area and sex.

Based on provincial patterns, the highest prevalence of being underweight was seen in the southeastern provinces (Data Sheets 1, 2). On the other hand, the highest prevalence of obesity belonged to the northeastern and central provinces (Figure 1 and Data Sheet 3).

The difference in age-standardized prevalence of obesity and overweight/obesity between the provinces were 17.9% (lowest: 11.7–highest: 29.6) and 28.1% (lowest: 38.8–highest: 66.9), respectively. The level of BMI increases as we move from the southeast to the northwest of the country (Data Sheet 4).

**Figure 2:**
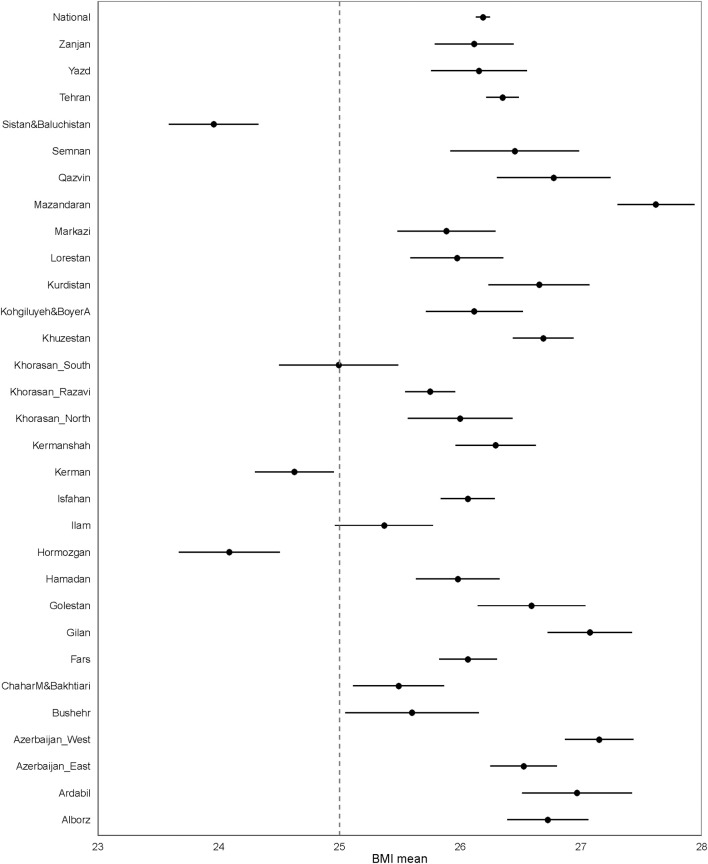
The national and provincial age-standardized mean BMI.

Considering the distribution of different categories of BMI across the provinces, similar to the national estimated level, the age-standardized mean BMI was >25 kg/m^2^ (Figure 2).

Sistan and Baluchistan had the highest underweight prevalence for both males (15.7%) and females (14.8%). It is noteworthy that in many provinces such as South Khorasan there is a double burden of obesity, especially among females (underweight prevalence is 12.1% and obesity prevalence is 19.5% for females) (Data Sheet 5).

Compared to rural areas, the mean BMI was significantly higher among individuals from urban areas for all age groups, in both males (Rural: 24.62, Urban: 26.02) and females (Rural: 26.74, Urban: 27.69) (Data Sheet 6).

The mean BMI was significantly higher in participants suffering from metabolic and lifestyle risk factors. A noteworthy point is that compared to the normal population, the mean BMI was significantly higher in patients with a positive history of heart attack and stroke over the past year (Table S1).

Among females, pre-diabetes based on FPG (OR: 1.71 [1.47–1.99]) or HbA1c (OR: 1.59 [1.37–1.83]), diabetes based on FPG (OR: 1.80 [1.44–2.26]) or HbA1c (OR: 1.90 [1.53–2.37]), LDL–C (OR: 1.48 [1.33–1.65]), hypertriglyceridemia (OR: 2.37 [2.05–2.75]), hypercholesterolemia (OR: 1.48 [1.27–1.71]), pre-hypertension (OR: 1.30 [1.20–1.41]), hypertension (OR: 1.96 [1.77–2.18]), and heart attack (OR: 1.81 [1.30–2.60]), increased the age-adjusted OR of overweight/obesity. Among males, pre-diabetes based on FPG (OR: 1.48 [1.29–1.71]) or HbA1c (OR: 1.31 [1.16–1.49]), diabetes based on FPG (OR: 2.01 [1.64–2.48]) or HbA1c (OR: 2.32 [1.90–2.84]), LDL–C (OR: 1.29 [1.16–1.43]), hypertriglyceridemia (OR: 3.33 [2.94–3.77]), hypercholesterolemia (OR: 1.90 [1.65–2.19]), pre-hypertension (OR: 1.31 [1.22–1.41]), hypertension (OR: 2.34 [2.12–2.57]), and heart attack (OR: 1.81 [1.30–2.60]), increased the age-adjusted OR of overweight/obesity. Ever tobacco smoking, ever daily cigarette smoking, and current daily cigarette smoking increased the age-adjusted OR of underweight and normal weight in both males and females and decreased the age-adjusted OR of overweight, overweight/obesity, and obesity (Table 3).

**Table 3:** The Odds Ratio of BMI categories according to metabolic and lifestyle risk factors of Iranian adults by sex.

**Variable**	**Sex**	**Status**	**Underweight** **(BMI < 18.5)**	**Normal** **(18.5 ≤ BMI < 25)**	**Overweight** **(25 ≤ BMI < 30)**	**Overweight/obesity** **(25 ≤ BMI)**	**Obesity** **(30 ≤ BMI)**	**Class I obesity** **(30 ≤ BMI < 35)**	**Class II obesity** **(35 ≤ BMI < 40)**	**Class III obesity** **(40 ≤ BMI)**
Pre-diabetes based on FPG (100 ≤ FPG < 126 mg/dl among who did not recognize as diabetic)	Female	No	1	1	1	1	1	1	1	1
		Yes	0.74 (0.51–1.08)	0.59 (0.50–0.69)^***^	0.93 (0.82–1.06)	1.71 (1.47–1.99)^***^	1.61 (1.41–1.83)^***^	1.29 (1.11–1.49)^**^	1.60 (1.30–1.98)^***^	2.05 (1.46–2.89)^***^
	Male	No	1	1	1	1	1	1	1	1
		Yes	0.58 (0.40–0.82)^**^	0.72 (0.62–0.83)^***^	1.18 (1.04–1.34)^*^	1.48 (1.29–1.71)^***^	1.40 (1.20–1.64)^***^	1.32 (1.11–1.56)^**^	1.61 (1.12–2.32)^*^	1.70 (0.79–3.65)
Pre-diabetes based on HbA1c (5.7 ≤ HbA1c < 6.4% among who did not recognize as diabetic)	Female	No	1	1	1	1	1	1	1	1
		Yes	0.78 (0.54–1.14)	0.63 (0.55–0.73)^***^	0.80 (0.70–0.90)^***^	1.59 (1.37–1.83)^***^	1.77 (1.57–2.01)^***^	1.42 (1.24–1.62)^***^	1.71 (1.38–2.12)^***^	1.96 (1.37–2.79)^***^
	Male	No	1	1	1	1	1	1	1	1
		Yes	1.11 (0.82–1.52)	0.74 (0.65–0.84)^***^	1.07 (0.95–1.21)	1.31 (1.16–1.49)^***^	1.38 (1.19–1.60)^***^	1.28 (1.09–1.51)^**^	1.64 (1.14–2.36)^**^	2.02 (0.91–4.49)
Diabetes based on FPG [FPG ≥ 126 mg/dl or self-report (OHA and/or insulin taking)]	Female	No	1	1	1	1	1	1	1	1
		Yes	0.10 (0.04–0.29)^***^	0.62 (0.49–0.78)^***^	0.95 (0.81–1.12)	1.80 (1.44–2.26)^***^	1.57 (1.33–1.85)^***^	1.41 (1.18–1.67)^***^	1.36 (1.05–1.75)^*^	1.40 (0.92–2.14)
	Male	No	1	1	1	1	1	1	1	1
		Yes	0.28 (0.14–0.55)^***^	0.56 (0.45–0.69)^***^	1.18 (0.98–1.42)	2.01 (1.64–2.48)^***^	1.99 (1.63–2.44)^***^	1.89 (1.52–2.34)^***^	1.77 (1.10–2.84)^*^	3.06 (1.22–7.70)^*^
Diabetes based on HbA1c [HbA1c ≥ 6.4% or self-report (OHA and/or insulin taking)]	Female	No	1	1	1	1	1	1	1	1
		Yes	0.10 (0.04–0.29)^***^	0.59 (0.47–0.73)^***^	0.98 (0.84–1.15)	1.90 (1.53–2.37)^***^	1.56 (1.33–1.83)^***^	1.38 (1.16–1.63)^***^	1.35 (1.06–1.73)^*^	1.57 (1.04–2.37)^*^
	Male	No	1	1	1	1	1	1	1	1
		Yes	0.19 (0.09–0.37)^***^	0.50 (0.41–0.61)^***^	1.31 (1.10–1.56)^**^	2.32 (1.90–2.84)^***^	2.04 (1.69–2.47)^***^	1.87 (1.53–2.29)^***^	1.98 (1.29–3.05)^**^	4.38 (1.94–9.86)^***^
LDL–C (low-density lipoprotein cholesterol ≥ 100 mg/dl)	Female	No	1	1	1	1	1	1	1	1
		Yes	0.46 (0.33–0.62)^***^	0.73 (0.65–0.81)^***^	1.11 (1.01–1.23)^*^	1.48 (1.33–1.65)^***^	1.26 (1.14–1.41)^***^	1.16 (1.04–1.31)^*^	1.22 (1.01–1.46)^*^	1.52 (1.11–2.09)^*^
	Male	No	1	1	1	1	1	1	1	1
		Yes	0.51 (0.39–0.67)^***^	0.85 (0.76–0.94)^**^	1.15 (1.03–1.27)^*^	1.29 (1.16–1.43)^***^	1.21 (1.06–1.39)^**^	1.21 (1.05–1.40)^**^	1.11 (0.79–1.56)	1.34 (0.67–2.68)
Hypertriglyceridemia (Triglyceride ≥ 150 mg/dl)	Female	No	1	1	1	1	1	1	1	1
		Yes	0.10 (0.05–0.21)^***^	0.47 (0.41–0.55)^***^	0.92 (0.82–1.04)	2.37 (2.05–2.75)^***^	2.04 (1.82–2.30)^***^	1.60 (1.41–1.82)^***^	2.08 (1.71–2.52)^***^	1.56 (1.11–2.19)^*^
	Male	No	1	1	1	1	1	1	1	1
		Yes	0.18 (0.11–0.28)^***^	0.35 (0.31–0.40)^***^	1.78 (1.59–1.98)^***^	3.33 (2.94–3.77)^***^	2.30 (2.01–2.63)^***^	2.19 (1.90–2.53)^***^	2.02 (1.48–2.77)^***^	2.93 (1.47–5.84)^**^
Hypercholesterolemia (total Cholesterol ≥ 200 mg/dl or self-report of drug taking)	Female	No	1	1	1	1	1	1	1	1
		Yes	0.34 (0.22–0.53)^***^	0.73 (0.63–0.85)^***^	1.08 (0.96–1.22)	1.48 (1.27–1.71)^***^	1.25 (1.11–1.41)^***^	1.26 (1.11–1.44)^**^	0.97 (0.79–1.19)	1.37 (0.93–2.03)
	Male	No	1	1	1	1	1	1	1	1
		Yes	0.30 (0.19–0.47)^***^	0.59 (0.51–0.68)^***^	1.28 (1.12–1.45)^***^	1.90 (1.65–2.19)^***^	1.72 (1.48–2.01)^***^	1.60 (1.36–1.88)^***^	1.78 (1.20–2.62)^**^	3.08 (1.47–6.46)^**^
Pre-hypertension (120 ≤ systolic blood pressure < 140 mmHg or 80 ≤ diastolic blood pressure < 90 mmHg among who did not recognize as hypertensive individual)	Female	No	1	1	1	1	1	1	1	1
		Yes	0.58 (0.47–0.73)^***^	0.83 (0.76–0.90)^***^	1.18 (1.09–1.27)^***^	1.30 (1.20–1.41)^***^	1.08 (0.99–1.17)	1.09 (1.00–1.19)	0.98 (0.84–1.13)	1.08 (0.85–1.39)
	Male	No	1	1	1	1	1	1	1	1
		Yes	0.59 (0.49–0.71)^***^	0.83 (0.77–0.89)^***^	1.30 (1.21–1.39)^***^	1.31 (1.22–1.41)^***^	1.03 (0.94–1.14)	1.06 (0.95–1.18)	0.98 (0.77–1.23)	0.67 (0.38–1.16)
Hypertension (systolic blood pressure ≥ 140 mmHg or diastolic blood pressure ≥ 90 mmHg or self-report of drug taking)	Female	No	1	1	1	1	1	1	1	1
		Yes	0.43 (0.32–0.58)^***^	0.54 (0.49–0.60)^***^	0.89 (0.81–0.97)^*^	1.96 (1.77–2.18)^***^	1.93 (1.76–2.12)^***^	1.53 (1.38–1.69)^***^	1.75 (1.50–2.05)^***^	2.49 (1.89–3.29)^***^
	Male	No	1	1	1	1	1	1	1	1
		Yes	0.38 (0.29–0.49)^***^	0.48 (0.44–0.53)^***^	1.34 (1.22–1.46)^***^	2.34 (2.12–2.57)^***^	2.34 (2.09–2.63)^***^	2.04 (1.81–2.31)^***^	2.57 (1.98–3.32)^***^	6.29 (3.44–11.52)^***^
Ever tobacco smoking	Female	No	1	1	1	1	1	1	1	1
		Yes	1.80 (1.34–2.43)^***^	1.18 (1.01–1.39)^*^	0.86 (0.74–0.99)^*^	0.76 (0.65–0.89)^**^	0.89 (0.76–1.04)	0.87 (0.74–1.03)	0.94 (0.69–1.27)	1.19 (0.78–1.83)
	Male	No	1	1	1	1	1	1	1	1
		Yes	1.83 (1.55–2.16)^***^	1.25 (1.16–1.35)^***^	0.79 (0.74–0.86)^***^	0.72 (0.67–0.78)^***^	0.83 (0.74–0.91)^***^	0.79 (0.71–0.88)^***^	1.08 (0.85–1.38)	0.86 (0.46–1.62)
Ever daily cigarette smoking	Female	No	1	1	1	1	1	1	1	1
		Yes	2.24 (1.29–3.87)^**^	1.56 (1.11–2.2)^*^	0.70 (0.52–0.94)^*^	0.57 (0.41–0.79)^**^	0.82 (0.61–1.12)	0.88 (0.64–1.21)	0.83 (0.41–1.66)	0.75 (0.30–1.87)
	Male	No	1	1	1	1	1	1	1	1
		Yes	1.89 (1.58–2.25)^***^	1.38 (1.28–1.50)^***^	0.75 (0.69–0.81)^***^	0.65 (0.60–0.70)^***^	0.76 (0.68–0.85)^***^	0.73 (0.65–0.82)^***^	1.01 (0.78–1.31)	0.90 (0.45–1.82)
Current daily cigarette smoking	Female	No	1	1	1	1	1	1	1	1
		Yes	3.24 (1.70–6.18)^***^	1.86 (1.27–2.73)^**^	0.70 (0.48–1.02)	0.44 (0.30–0.64)^***^	0.60 (0.40–0.91)^*^	0.71 (0.45–1.12)	0.43 (0.18–1.07)	0.96 (0.30–3.08)
	Male	No	1	1	1	1	1	1	1	1
		Yes	1.95 (1.61–2.36)^***^	1.57 (1.44–1.72)^***^	0.70 (0.64–0.77)^***^	0.57 (0.52–0.62)^***^	0.65 (0.57–0.74)^***^	0.61 (0.53–0.70)^***^	0.94 (0.71–1.26)	0.89 (0.40–1.98)
Heart attack incidence within the last year (self-report)	Female	No	1	1	1	1	1	1	1	1
		Yes	1.23 (0.60–2.60)	0.50 (0.30–0.70)^**^	0.87 (0.60–1.20)	1.81 (1.30–2.60)^**^	1.93 (1.40–2.60)^***^	1.45 (1.00–2.10)^*^	2.00 (1.20–3.20)^**^	2.32 (1.10–4.90)^*^
	Male	No	1	1	1	1	1	1	1	1
		Yes	0.51 (0.20–1.20)	0.53 (0.40–0.70)^***^	1.22 (0.90–1.60)	2.03 (1.50–2.70)^***^	2.07 (1.50–2.80)^***^	1.73 (1.30–2.40)^**^	3.24 (1.90–5.50)^***^	0.94 (0.10–6.80)
Stroke incidence within the last year (self-report)	Female	No	1	1	1	1	1	1	1	1
		Yes	0.91 (0.30–2.50)	0.65 (0.40–1.10)	0.71 (0.40–1.10)	1.51 (1.00–2.40)	2.00 (1.30–3.00)^**^	1.69 (1.10–2.60)^*^	1.37 (0.70–2.80)	3.12 (1.30–7.30)^**^
	Male	No	1	1	1	1	1	1	1	1
		Yes	0.49 (0.10–2.00)	0.97 (0.60–1.50)	0.86 (0.60–1.30)	1.12 (0.70–1.70)	1.53 (0.90–2.50)	1.09 (0.60–2.00)	2.36 (0.90–5.90)	7.04 (2.10–23.30)^**^

*Data in parentheses are 95% Confidence Intervals (CI)*.

* Significant at p < 0.05;

** Significant at p < 0.01;

*** *Significant at p < 0.001*.

## Discussion

We estimated the prevalence of overweight/obesity and distribution of BMI levels in the Iranian population, by sex, age, and geographical distribution. Our findings show that, in 2016, approximately 22.7% (95% CI: 22.2–23.2) of the 18≤ year-old Iranian adults were obese and 59.3% (58.7–59.9) were overweight. We found a significant difference between the prevalence of obesity in males and females. The highest provincial prevalence of obesity (29.6% for Mazandaran) was almost 2.5 times higher than the lowest provincial prevalence of obesity (11.7% for Hormozgan).

The number of obese and overweight people has become an important health concern in many developing countries, however, the predisposing factors and affiliated adverse health outcomes follow different patterns in different populations (1, 15). In 2014, in Iran, the global age-standardized mean BMI in males and females was estimated at 24.2 (24.0–24.4) kg/m^2^ and 24.4 (24.2–24.6) kg/m^2^, respectively (1). Age-standardized prevalence of obesity was estimated at 10.8% (9.7–12.0) in males, and 14.9% (13.6–16.1) in females. At the same time, 2.3% (2.0–2.7) of males and 5.0% (4.4–5.6) of females were severely obese (BMI ≥ 35 kg/m^2^) at global level. The age-standardized global prevalence of underweight was 8.8% (7.4–10.3) in males and 9.7% (8.3–11.1) in females (1).

Several studies have provided reports on obesity trends in Iran and these trends mostly match our findings (1, 3, 6, 15). The Global Burden of Diseases (GBD) study has reported the obesity prevalence in Iranian adult females and males (≥20 years) at 29.3 and 13.6%, respectively; significantly lower than our estimates (3). Considering the multifactorial nature of obesity, in order to investigate the causes of change and reported values, both medical and non-medical predisposing factors such as age, gender, race/ethnicity, socioeconomic status, and- understandably- lifestyle patterns have been discussed (1, 16). Moreover, given the effects of epidemiological changes, many sources of differences can be extracted from data quality and our approaches toward applying data-driven estimates or relying on the estimates that were derived from different modeling methods (1, 9).

Considering gender differences, studies in many regions of the world have shown that, compared to males, females are at greater risk of obesity (15, 17). Evidence has confirmed that males and females have differences in anatomical fat distribution, fat utilization, and obesity co-morbidities (18). These may be rooted in differences in genetics, sex hormones, and even unknown molecular mechanisms (17, 18).

Geographically, the highest levels of BMI were detected in the northwestern and central provinces. Earlier studies have shown that these regions have a mostly higher economic status (5). Results from other relevant research indicate that during recent decades, there has been a slower increase in BMI in high-income populations (1, 19). On the other hand, some studies have shown otherwise. For instance, in a comprehensive analysis of global data, the largest increase in BMI occurred in high-income English-speaking countries (1). In this regard, numerous components such as variations in lifestyle and geographical factors have been discussed in the literature (1, 17, 20).

The current paper brings to light the most updated results of overweight/obesity and BMI at national and subnational levels in 2016. Using proportional to size sampling, one of the main strengths of the study was its comprehensive protocols focused on standards and regulatory guidelines. Moreover, the digitalized online study provided the most reliable data that could be used as practical evidence for better policymaking.

We also faced many limitations. The main limitation of this study is its cross-sectional nature, which limits us in the inferential analysis. Data from previous rounds of the STEPs surveys are available and might be the subject of another study using meta-regression to arrive at better estimates of the BMI or obesity trends at national and subnational levels (21, 22).

The present study has many important implications. Based on other countries' experiences, the current ongoing interventions and policies are not enough to stop the rise in BMI (1, 11, 17, 23).

The global NCD target of obesity cleared the path for policies on the worldwide management of the problem. Based on evidence, we must exactly follow our defined national goals (11, 24). As the next step, monitoring and evaluation of the implemented programs must be investigated carefully. For better planning and more effective interventions, we need comprehensive approaches benefiting from all national resources and capacities. In this regard, literature confirms that primary care systems with trained community health-care workers together with well-developed guidelines can be effective in preventing and managing non-communicable diseases and risk factors (2, 23, 25). Moreover, a wide range of interventions for prevention or treatment may be selected based on comprehensive plans that involve different aspects of individual and population interventions aimed at specific target groups (16, 26). Given the gaps in the relevant evidence needed, more research on individual and social obesity-related behaviors should be conducted through complementary studies (16, 26).

Another important point is that, like many other countries that have focused on obesity and its adverse health outcomes, the issue and consequences of being underweight in many subpopulations has largely been overshadowed in Iran too (27, 28). The alarming burden caused by malnutrition in females of reproductive age, pregnant females, and children, must lead to interventions focused on prompt problem-based solutions (27, 28). In order to manage the problem, social and food policies must include practical policies and strategies that enhance food security, especially among poor households. Furthermore, the prevention of overconsumption of unhealthy foods such as processed carbohydrates must also be borne in mind (29). Along with these considerations, the screening of at-risk populations, detection of possible comorbidities (such as anorexia nervosa), nutritional supplementation in pregnant females and students, and using the most effective and safe treatment for weight restoration in inpatient target groups, should be undertaken as appropriate population-based interventions (19, 30).

Following the global agenda on risk reduction, with the participation of all stakeholders, Iran developed a comprehensive national non-communicable diseases action plan through which prevention and control of overweight and obesity were targeted as the main risk factors. Benefiting from a multisectoral approach, supplementary agreements were signed for the implementation of obligations on behalf of other collaborating organizations (24, 31).

It is also remarkable that, in spite of considerable earlier efforts, there are still noticeable gaps and limitations in the evidence required for policy making. The possible causes of patterns of risk factors as well as the epidemiological transition should be investigated for various metabolic risks and all risks in more detail. Moreover, behavioral changes in smoking, physical activity, alcohol intake, and psychosocial factors according to demographic specifications such as sex, age, and ethnicity, must be addressed further as a complex set of predisposing factors (1, 6, 15, 28). These should be followed through complementary researches in relevant and specific fields.

## Conclusion

In conclusion, to the best of our knowledge, the present study is the first comprehensive experience of a systematic and fully digitalized national survey in Iran. Given the evidence on national and subnational requirements for the promotion of strategies and interventions to prevent and control weight gain, and to achieve SDG 3.4 and the WHO Action Plan for NCD control and prevention, we propose comprehensive programs that meet the needs of all stakeholders. We found a significant difference between the prevalence of obesity in males and females. Moreover, there was a considerable difference in the geographical pattern of the prevalence of obesity and overweight. Further focus needs to be laid on trends analyses of BMI risk factors to identify the priority interventions.

## Data Availability Statement

The data used in the current study are available upon request from the corresponding author. Aggregated reports are published online on https://vizit.report and are freely available to public for non-commercial use.

## Author Contributions

FF and SD: general design of paper. FF, SS, AS, and SD: design of methods. FF, AS, SS, MY, and KG: analysis. SD, SS, AS, and SSN: primary drafting of the manuscript. BL, MM, HZ, MY, KG, NR, SN, AK, ZA, and ARS: manuscript revision.

### Conflict of Interest

The authors declare that the research was conducted in the absence of any commercial or financial relationships that could be construed as a potential conflict of interest.

## Footnotes

^1^https://www.amar.org.ir/english/Population-and-Housing-Censuses

## Supplementary Material

Data Sheet 1 The provincial distribution of age-standardized prevalence of underweight (%) by residential area and sex.

Data Sheet 2 The provincial distribution of age-standardized prevalence of normal weight (%) by residential area and sex.

Data Sheet 3 The provincial distribution of age-standardized prevalence of overweight/obesity (%) by residential area and sex.

Data Sheet 4 The provincial distribution of age-standardized mean BMI by residential area and sex.

Data Sheet 5 Distribution of the BMI categories' percentage by sex and province.

Data Sheet 6 Distribution of the mean BMI and its categories by sex, residential area, and age groups.

Table S1 The BMI mean according to metabolic and lifestyle risk factors by sex in Iranian adults.
