# Supplementary material for: Patterns of Obesity and Overweight in the Iranian Population: Findings of STEPs 2016
Source: Front Endocrinol (Lausanne). 2020 Feb 26;11:42. doi: 10.3389/fendo.2020.00042 (PMC7055062; doi:10.3389/fendo.2020.00042)
Supplement: Data Sheet 1 — The provincial distribution of age-standardized prevalence of underweight (%) by residential area and sex. [file Data_Sheet_1.PDF]

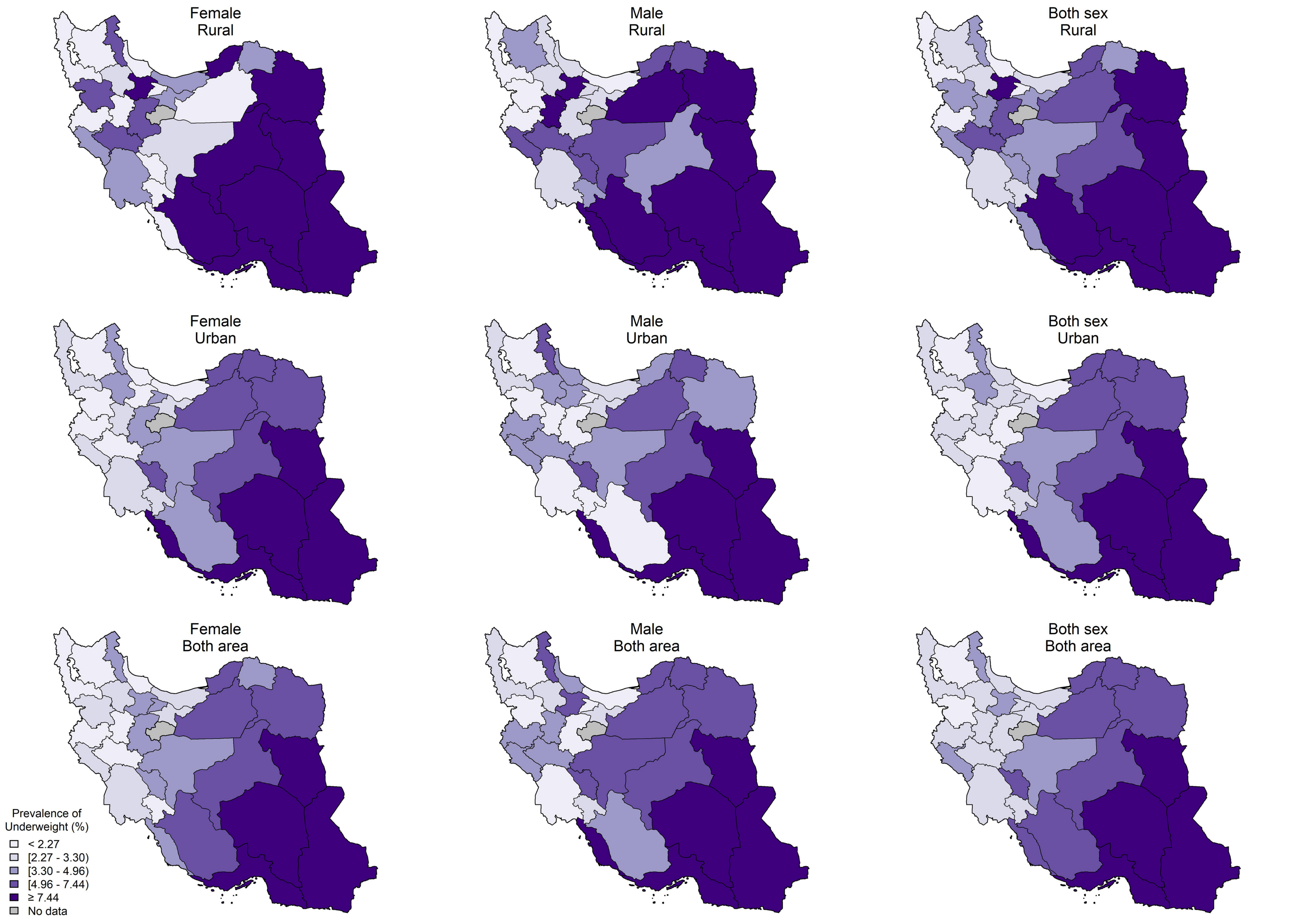
Female
Rural
Male
Rural
Both sex
Rural
Female
Urban
Male
Urban
Both sex
Urban
Female
Both area
Male
Both area
Both sex
Both area
Prevalence of
Underweight (%)
< 2.27
[2.27 - 3.30)
[3.30 - 4.96)
[4.96 - 7.44)
≥ 7.44
No data
