# Supplementary material for: Patterns of Obesity and Overweight in the Iranian Population: Findings of STEPs 2016
Source: Front Endocrinol (Lausanne). 2020 Feb 26;11:42. doi: 10.3389/fendo.2020.00042 (PMC7055062; doi:10.3389/fendo.2020.00042)
Supplement: Data Sheet 2 — The provincial distribution of age-standardized prevalence of normal weight (%) by residential area and sex. [file Data_Sheet_2.PDF]

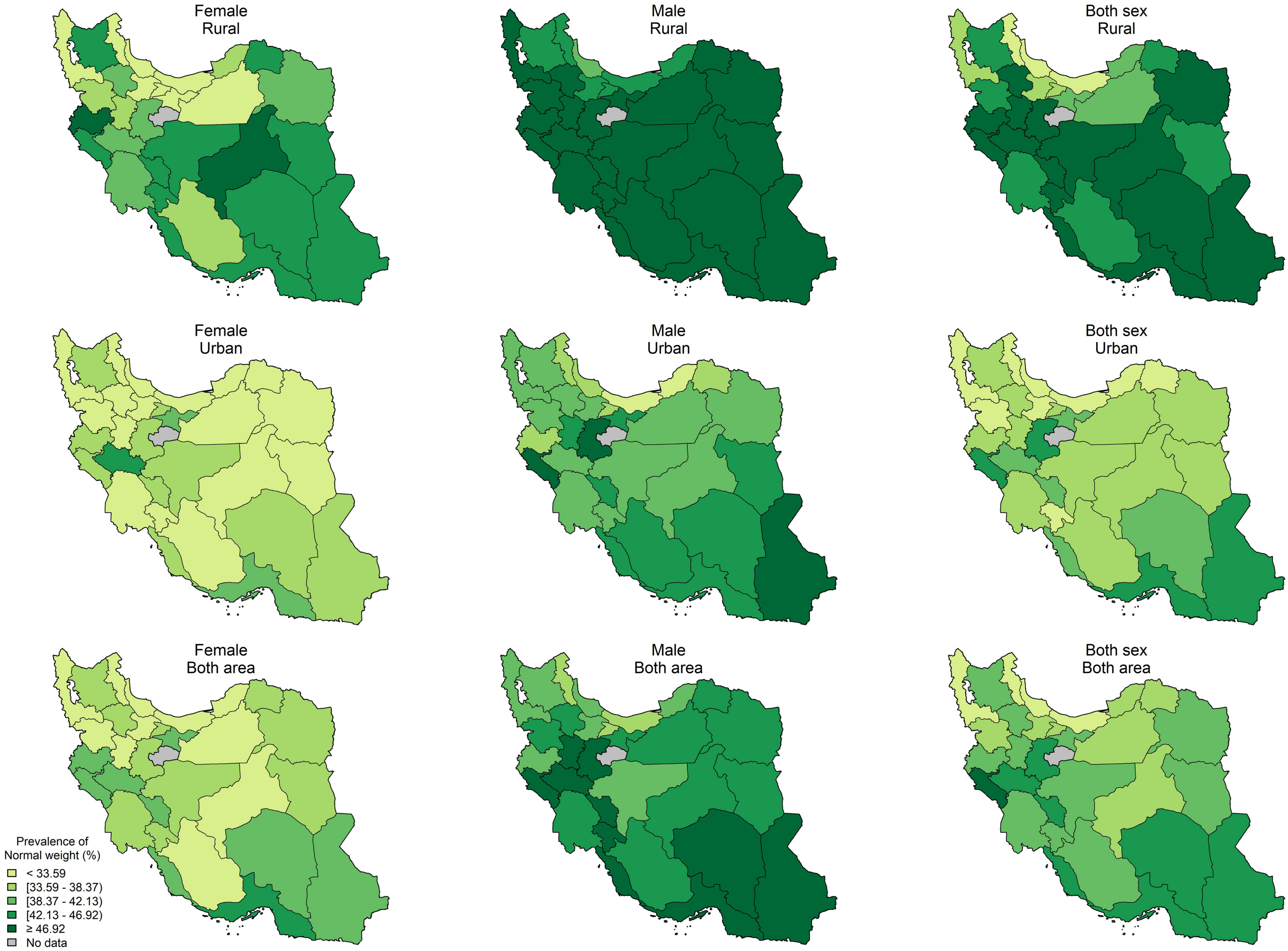
Female
Rural
Male
Rural
Both sex
Rural
Female
Urban
Male
Urban
Both sex
Urban
Female
Both area
Male
Both area
Both sex
Both area
Prevalence of
Normal weight (%)
< 33.59
[33.59 - 38.37)
[38.37 - 42.13)
[42.13 - 46.92)
≥ 46.92
No data
