# Supplementary material for: Patterns of Obesity and Overweight in the Iranian Population: Findings of STEPs 2016
Source: Front Endocrinol (Lausanne). 2020 Feb 26;11:42. doi: 10.3389/fendo.2020.00042 (PMC7055062; doi:10.3389/fendo.2020.00042)
Supplement: Data Sheet 3 — The provincial distribution of age-standardized prevalence of overweight/obesity (%) by residential area and sex. [file Data_Sheet_3.PDF]

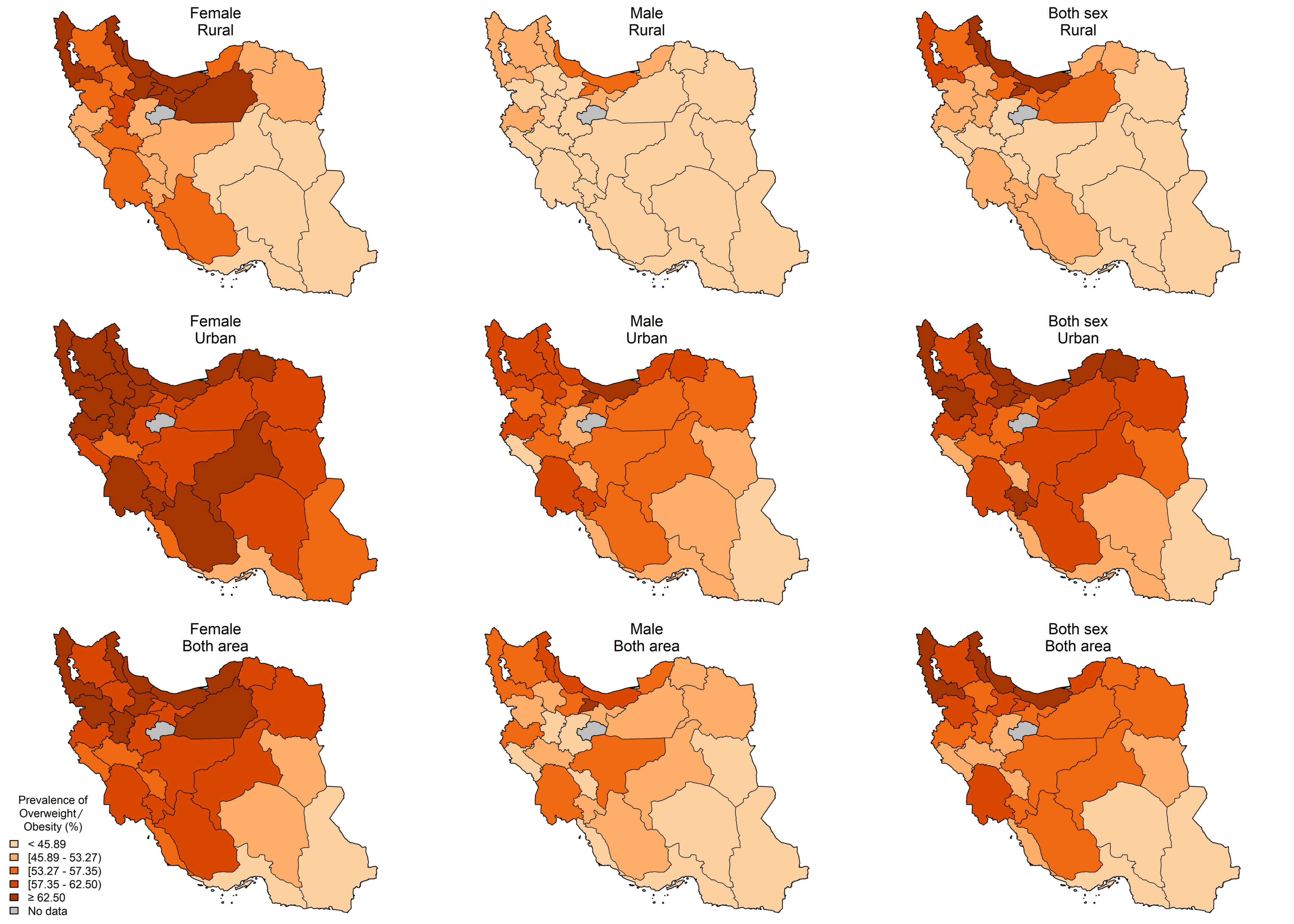

Female
Rural
Male
Rural
Both sex
Rural
Female
Urban
Male
Urban
Both sex
Urban
Female
Both area
Male
Both area
Both sex
Both area
Prevalence of Overweight / Obesity (%)
< 45.89
[45.89 - 53.27)
[53.27 - 57.35)
[57.35 - 62.50)
≥ 62.50
No data
