# Supplementary material for: Patterns of Obesity and Overweight in the Iranian Population: Findings of STEPs 2016
Source: Front Endocrinol (Lausanne). 2020 Feb 26;11:42. doi: 10.3389/fendo.2020.00042 (PMC7055062; doi:10.3389/fendo.2020.00042)
Supplement: Data Sheet 4 — The provincial distribution of age-standardized mean BMI by residential area and sex. [file Data_Sheet_4.PDF]

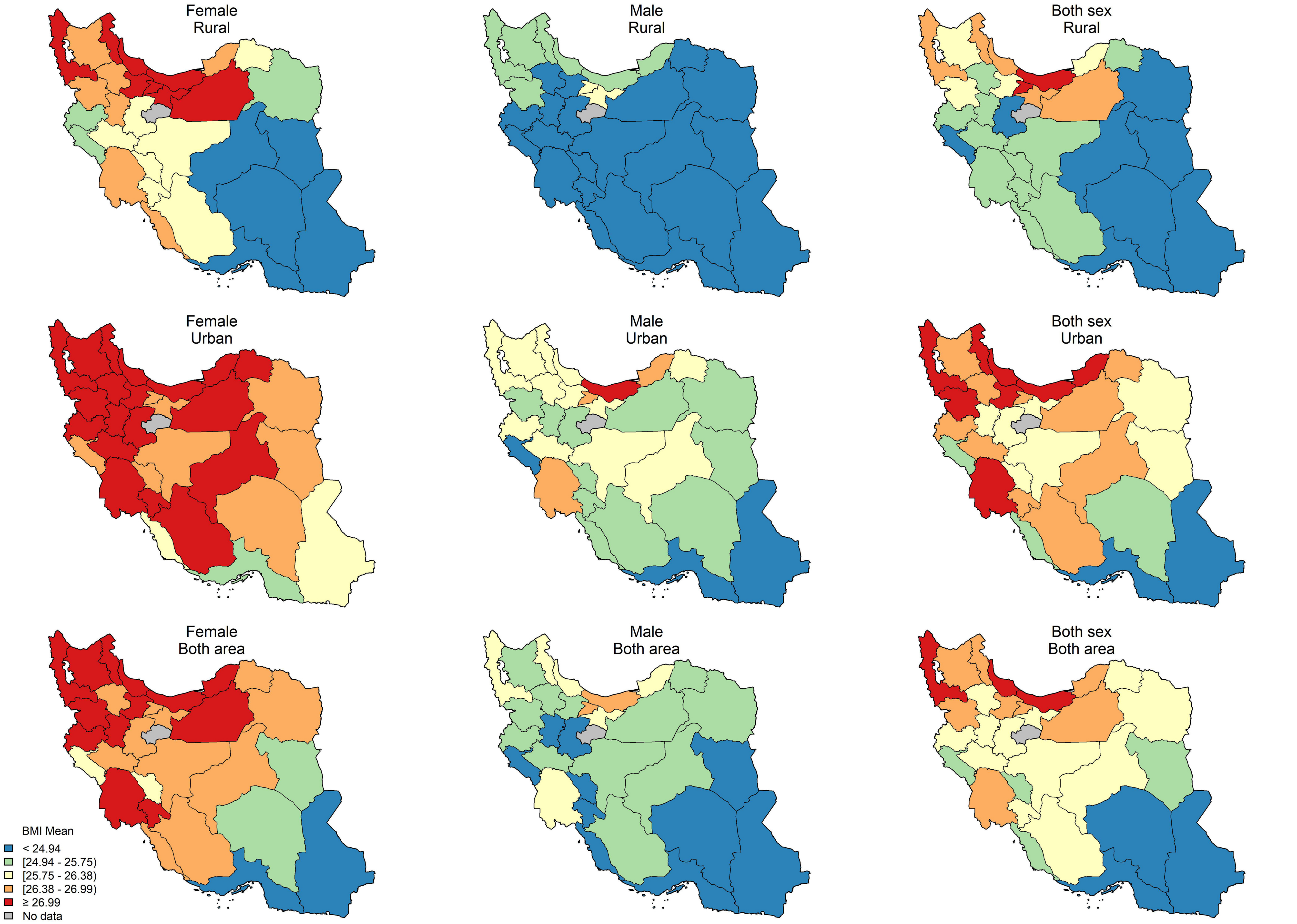
Female
Rural
Male
Rural
Both sex
Rural
Female
Urban
Male
Urban
Both sex
Urban
Female
Both area
Male
Both area
Both sex
Both area
BMI Mean
< 24.94
[24.94 - 25.75)
[25.75 - 26.38)
[26.38 - 26.99)
≥ 26.99
No data
