# Supplementary figures and images for: Patterns of Obesity and Overweight in the Iranian Population: Findings of STEPs 2016
Source: Front Endocrinol (Lausanne). 2020 Feb 26;11:42. doi: 10.3389/fendo.2020.00042 (PMC7055062; doi:10.3389/fendo.2020.00042)

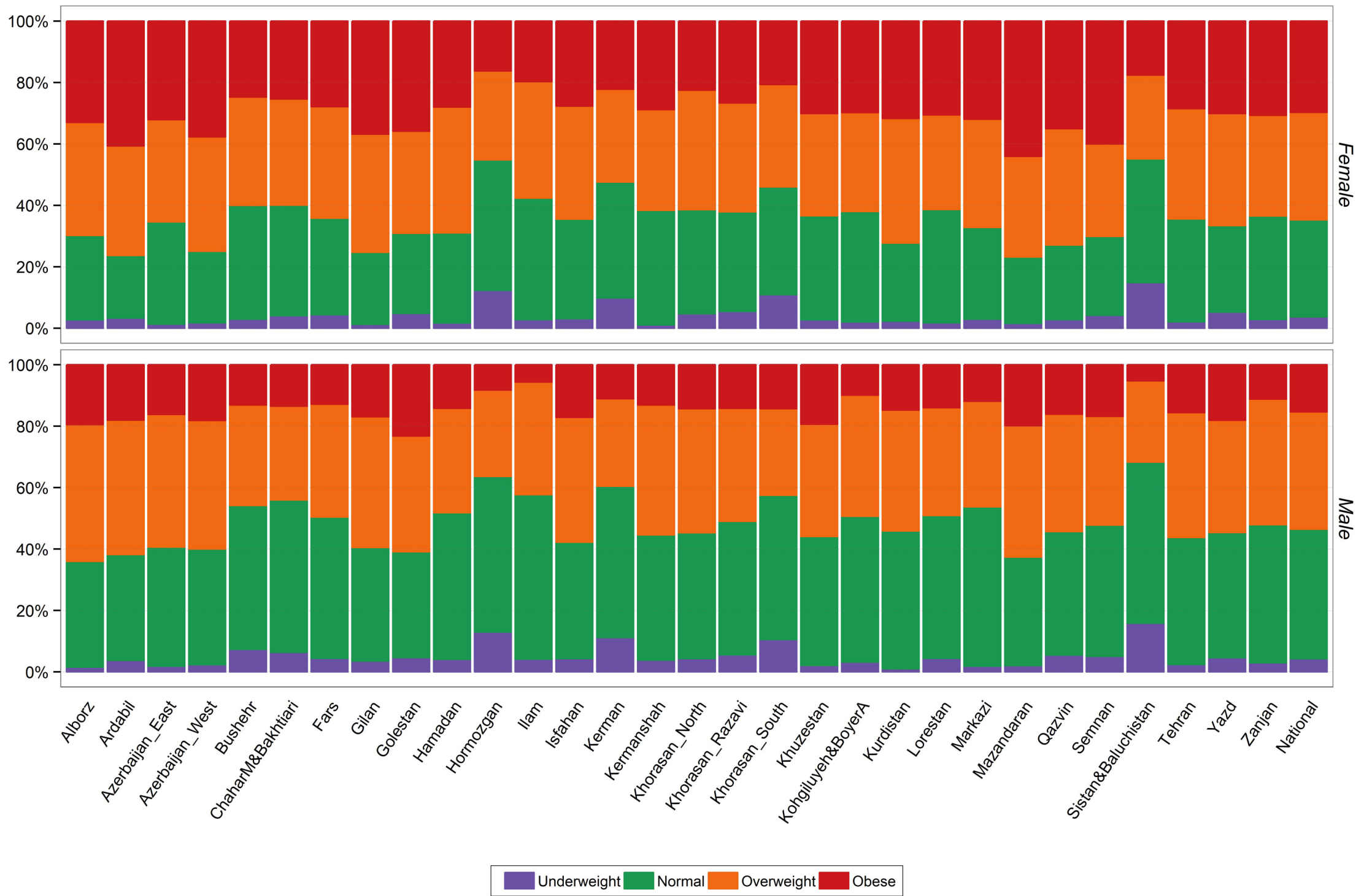

Supplement: Data Sheet 5 — Distribution of the BMI categories' percentage by sex and province. [file Data_Sheet_5.PDF]

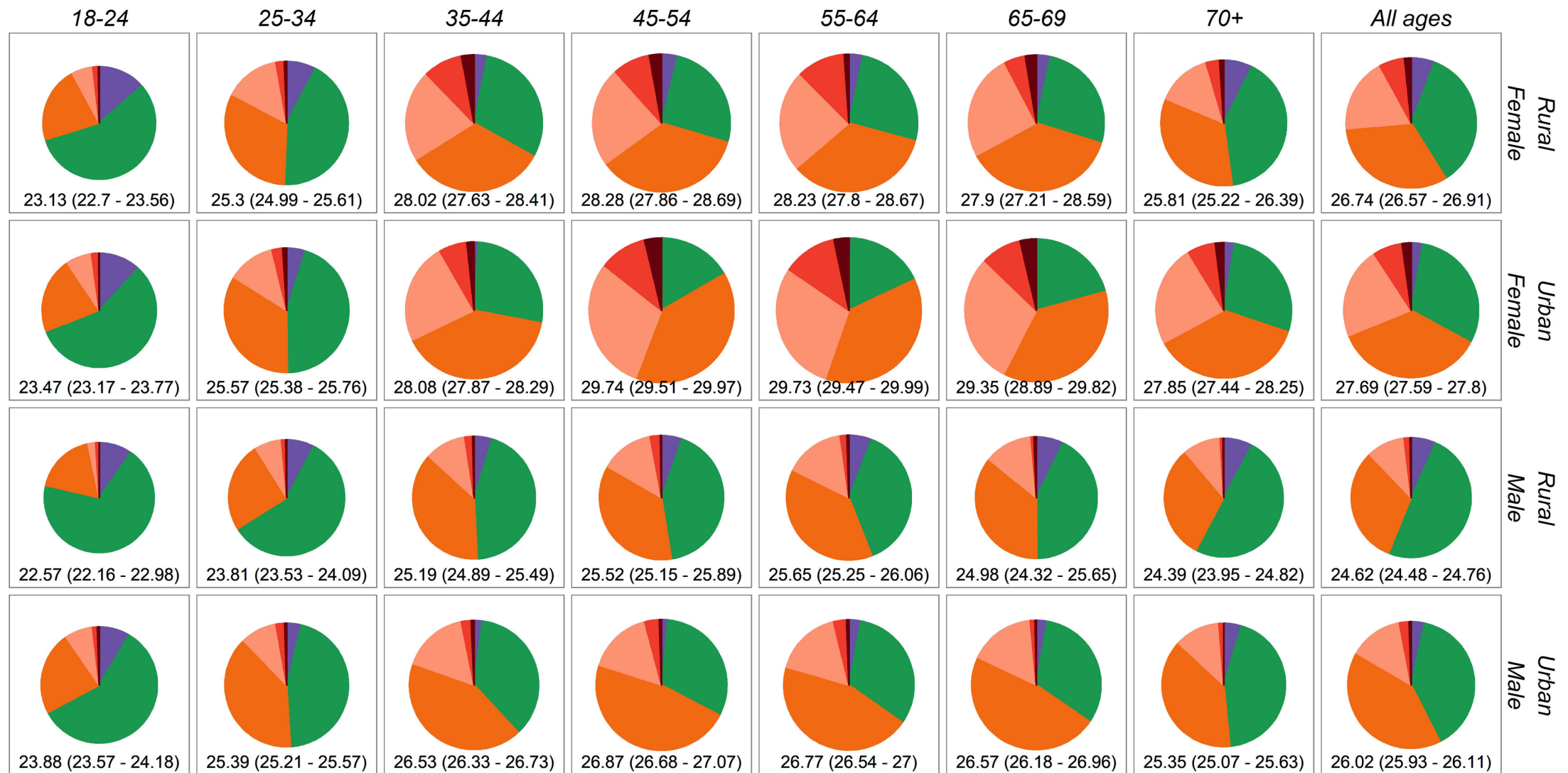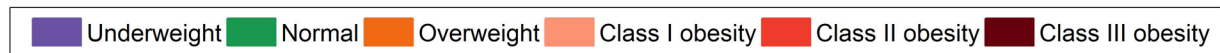

Supplement: Data Sheet 6 — Distribution of the mean BMI and its categories by sex, residential area, and age groups. [file Data_Sheet_6.PDF]
