# Supplementary material for: Patterns of Obesity and Overweight in the Iranian Population: Findings of STEPs 2016
Source: Front Endocrinol (Lausanne). 2020 Feb 26;11:42. doi: 10.3389/fendo.2020.00042 (PMC7055062; doi:10.3389/fendo.2020.00042)
Supplement: Table S1 — The BMI mean according to metabolic and lifestyle risk factors by sex in Iranian adults. [file Table_1.docx]

Supplementary Table 1: The BMI mean according to metabolic and lifestyle risk factors by sex in Iranian adults

| ***Variable*** | ***Status*** | ***BMI mean (95% CI)*** | | |
| --- | --- | --- | --- | --- |
|  |  | ***Female*** | ***Male*** | ***Both*** |
| ***Overall*** | | 27.4 (27.3-27.5) | 25.6 (25.5-25.7) | 26.5 (26.5-26.6) |
| ***Pre-diabetes based on FPG (100≤FPG<126 mg/dl among who did not recognize as diabetic)*** | ***No*** | 27.7 (27.6-27.9) | 25.7 (25.6-25.8) | 26.8 (26.7-26.9) |
|  | ***Yes*** | 29.7 (29.4-30.0) | 26.9 (26.7-27.2) | 28.4 (28.1-28.6) |
| ***Pre-diabetes based on HbA1c (5.7≤HbA1c<6.4% among who did not recognize as diabetic)*** | ***No*** | 27.6 (27.5-27.7) | 25.8 (25.7-25.9) | 26.8 (26.7-26.9) |
|  | ***Yes*** | 29.7 (29.5-30.0) | 25.8 (25.7-25.9) | 28.2 (28.1-28.4) |
| ***Diabetes based on FPG***  ***(FPG≥126 mg/dl or self-report (OHA and/or Insulin taking))*** | ***No*** | 27.8 (27.7-28.0) | 25.8 (25.7-25.9) | 26.9 (26.8-27.0) |
|  | ***Yes*** | 29.9 (29.6-30.3) | 27.6 (27.2-27.9) | 28.9 (28.7-29.2) |
| ***Diabetes based on HbA1c***  ***(HbA1c≥6.4% or self-report (OHA and/or Insulin taking))*** | ***No*** | 27.8 (27.7-27.9) | 25.7 (25.6-25.8) | 26.8 (26.8-26.9) |
|  | ***Yes*** | 30.0 (29.7-30.3) | 27.7 (27.4-28.0) | 29.0 (28.8-29.3) |
| ***LDL–C***  ***(Low-density lipoprotein cholesterol≥100 mg/dl)*** | ***No*** | 27.4 (27.3-27.6) | 25.6 (25.4-25.7) | 26.6 (26.4-26.7) |
|  | ***Yes*** | 28.9 (28.7-29.0) | 26.5 (26.3-26.6) | 27.8 (27.7-27.9) |
| ***Hypertriglyceridemia***  ***(Triglyceride≥150 mg/dl)*** | ***No*** | 27.4 (27.3-27.6) | 25.1 (25.0-25.2) | 26.4 (26.3-26.5) |
|  | ***Yes*** | 30.1 (29.9-30.4) | 27.8 (27.6-28.0) | 28.9 (28.8-29.1) |
| ***Hypercholesterolemia***  ***(Total Cholesterol≥200 mg/dl or self-report of drug taking)*** | ***No*** | 27.7 (27.6-27.9) | 25.6 (25.5-25.7) | 26.7 (26.6-26.8) |
|  | ***Yes*** | 29.2 (29.0-29.5) | 27.4 (27.2-27.6) | 28.5 (28.3-28.7) |
| ***Pre-hypertension***  ***(120≤Systolic blood pressure<140 mmHg or 80≤Diastolic blood pressure<90 mmHg among who did not recognize as hypertensive individual)*** | ***No*** | 27.2 (27.1-27.3) | 25.4 (25.3-25.5) | 26.4 (26.3-26.5) |
|  | ***Yes*** | 27.9 (27.8-28.1) | 25.9 (25.8-26.0) | 26.8 (26.7-26.9) |
| ***Hypertension***  ***(Systolic blood pressure≥140 mmHg or Diastolic blood pressure≥90 mmHg or self-report of drug taking)*** | ***No*** | 26.6 (26.5-26.7) | 25.1 (25.0-25.2) | 25.9 (25.8-25.9) |
|  | ***Yes*** | 29.6 (29.4-29.7) | 27.2 (27.1-27.4) | 28.5 (28.4-28.6) |
| ***Ever tobacco smoking*** | ***No*** | 27.4 (27.3-27.5) | 25.8 (25.7-25.9) | 26.8 (26.7-26.9) |
|  | ***Yes*** | 27.2 (26.8-27.6) | 25.3 (25.2-25.4) | 25.6 (25.5-25.7) |
| ***Ever daily cigarette smoking*** | ***No*** | 27.4 (27.3-27.5) | 25.8 (25.7-25.8) | 26.7 (26.7-26.8) |
|  | ***Yes*** | 27.3 (26.4-28.1) | 25.2 (25.1-25.4) | 25.4 (25.2-25.5) |
| ***Current daily cigarette smoking*** | ***No*** | 27.4 (27.3-27.5) | 25.8 (25.7-25.9) | 26.7 (26.7-26.8) |
|  | ***Yes*** | 26.4 (25.3-27.4) | 24.9 (24.8-25.1) | 25.0 (24.8-25.2) |
| ***Heart attack incidence within the last year***  ***(Self-report)*** | ***No*** | 27.4 (27.3-27.5) | 25.6 (25.5-25.7) | 26.5 (26.5-26.6) |
|  | ***Yes*** | 29.3 (28.3-30.2) | 27.6 (27.0-28.1) | 28.2 (27.7-28.8) |
| ***Stroke incidence within the last year***  ***(Self-report)*** | ***No*** | 27.4 (27.3-27.5) | 25.6 (25.5-25.7) | 26.5 (26.5-26.6) |
|  | ***Yes*** | 29.5 (28.2-30.7) | 26.7 (25.6-27.8) | 28.1 (27.2-28.9) |

*Data in parentheses are 95% Confidence Intervals (CI)*
